# Physician perspectives on chronic pain management: barriers and the use of eHealth in the COVID-19 era

**DOI:** 10.1186/s12913-023-10157-8

**Published:** 2023-10-20

**Authors:** Kimberley Kaseweter, Mark Nazemi, Nina Gregoire, W. Francois Louw, Zach Walsh, Susan Holtzman

**Affiliations:** 1 Department of Psychology, University of British Columbia, 3333 University Way, Kelowna, BC V1V 1V7 Canada; 2 Clinical and Wellbeing Solutions, Thrive Health Inc, 200 - 116 West Hastings Street, Vancouver, BC V6B 1G8 Canada; 3 Department of Family Practice, University of British Columbia, Vancouver, BC V6T 1Z4 Canada; 4 Bill Nelems Pain and Research Centre, 309-2755 Tutt St, Kelowna, BC V1Y 0G1 Canada

**Keywords:** Chronic pain, Health care delivery, Barriers, Access to care, Physician perspectives, Preferences, eHealth technology, Primary care, COVID-19

## Abstract

**Background:**

Chronic pain is a highly prevalent and disabling condition which is often undertreated and poorly managed in the community. The emergence of COVID-19 has further complicated pain care, with an increased prevalence of chronic pain and mental health comorbidities, and burnout among physicians. While the pandemic has led to a dramatic increase in virtual health care visits, the uptake of a broader range of eHealth technologies remains unclear. The present study sought to better understand physicians’ current needs and barriers in providing effective pain care within the context of COVID-19, as well as gauge current use, interest, and ongoing barriers to eHealth implementation.

**Methods:**

A total of 100 practicing physicians in British Columbia, Canada, completed a brief online survey.

**Results:**

The sample was comprised of physicians practicing in rural and urban areas (rural = 48%, urban = 42%; both = 10%), with the majority (72%) working in family practice. The most prominent perceived barriers to providing chronic pain care were a lack of interdisciplinary treatment and allied health care for patients, challenges related to opioid prescribing and management, and a lack of time to manage the complexities of chronic pain. Moreover, despite expressing considerable interest in eHealth for chronic pain management (82%), low adoption rates were observed for several technologies. Specifically, only a small percentage of the sample reported using eHealth for the collection of intake data (21%), patient-reported outcomes (14%), and remote patient monitoring (26%). The most common perceived barriers to implementation were cost, complexity, and unfamiliarity with available options.

**Conclusions:**

Findings provide insight into physicians’ ongoing needs and barriers in providing effective pain management during the COVID-19 pandemic. Despite the potential for eHealth technologies to help address barriers in pain care, and strong interest from physicians, enhanced useability, education and training, and funding are likely required to achieve successful implementation of a broader range of eHealth technologies in the future.

## Introduction

Chronic pain has been identified as the primary cause of disability worldwide [1]. Prevalence rates of chronic pain range from 11 to 40% globally [2], and in Canada, chronic pain affects an estimated one in four individuals [3, 4]. Compared to patients with other long-term illnesses (e.g., diabetes, coronary artery disease), people with chronic pain have significantly lower quality of life scores across all health-related domains, including physical, social, and emotional functioning [5, 6]. In addition to contributing to patient suffering, chronic pain costs Canada $56 billion annually, in both direct (e.g., medications, provider fees) and indirect (e.g., missed work) expenses [7].

Chronic pain has a long history of being undertreated and inadequately managed in Canada [8–12] and globally [2]. One factor complicating chronic pain management is the multidimensional nature of the condition, which often manifests with complex physical and psychological comorbidities (e.g., depression and insomnia) [13, 14]. As such, interdisciplinary pain clinics, which offer integrated services that target the biological and psychosocial factors underlying patients’ pain, have become the gold standard in chronic pain treatment [15, 16]. Specifically, interdisciplinary care is a healthcare model in which multiple healthcare professionals with different specialties, including physicians, nurses, social workers, pharmacists, and mental health therapists work collaboratively as a team to provide personalized, coordinated, integrated, and patient-centered care [17]. By bringing together their unique perspectives and expertise, the healthcare providers aim to address the patient’s physical, emotional, social, and psychological needs, with the ultimate goal of improving patient outcomes through coordinated and comprehensive care. However, access to these clinics is greatly restricted due to a limited number of facilities and long waitlists [5, 7]. Recent Canadian data also highlights that clinic exclusion criteria disproportionately impacts patients with complex pain issues (e.g., fibromyalgia, migraines) and psychosocial comorbidities (e.g., substance use, mental health disorders), leaving many vulnerable individuals without access to specialist care [18]. Further hindering an interdisciplinary pain care approach is the lack of public funding for community-based allied health services, such as psychological therapy and physiotherapy, leaving these services often unaffordable for patients [19–21].

Consequently, the vast majority of patients with chronic pain are cared for in the context of primary care settings, such as family practices and walk-in clinics [22–24]. In addition, these patients tend to see their family physician twice as often as other patients and typically require longer appointments [14, 25–27]. This has placed a heavy demand on family physicians, who often report inadequate pain education and low confidence in their ability to manage chronic pain effectively [28–32]. Perhaps unsurprisingly, recent studies suggest that many physicians perceive the treatment of chronic pain as uniquely challenging, conveying feelings of exhaustion, frustration, and reduced job satisfaction [33, 34]. These issues have been further compounded by the COVID-19 pandemic, with physicians facing even greater workloads and burnout [35–37]. Moreover, COVID-19 has contributed to an increased prevalence of chronic pain and an exacerbation of pain symptoms and comorbidities [38–40]. As a result, patients are failing to receive care due to higher clinic volumes, resource reallocation, and restricted services [41, 42].

Among the many potential strategies for improving pain care is a greater uptake and diversification of the use of eHealth platforms to assist physicians in the assessment and treatment of chronic pain in their daily practices [41, 43–45]. Broadly speaking, eHealth platforms refer to internet-based technologies applied in the context of healthcare [46]. Such platforms include web-based technologies that collect patient-reported data, provide clinician decision-support, and facilitate virtual visits, remote patient monitoring, and specialist consults [47–50]. Prior to the COVID-19 pandemic, the implementation of eHealth technologies into routine care was proving difficult, with low adoption rates often observed in clinical practice [51–54]. Challenges with integration were often attributed to a greater perceived workload, lack of eHealth awareness and training, inadequate funding to support implementation and sustainability, and low perceptions of useability and utility [51, 52, 55–57]. However, the onset of the COVID-19 pandemic necessitated a widespread, global adoption of virtual care [58]. For instance, a recent Canadian study found that the percentage of in-office visits fell by 79.1%, and virtual visits rose 56-fold during the pandemic, resulting in 71.1% of primary care physician visits being held virtually [58]. While there is strong evidence of implementation of virtual visits, physician uptake of other forms of eHealth technology, such as online patient intakes and remote patient monitoring, as well as persistent barriers to such uptake, remains less clear.

### Present Study

The present study was designed to achieve two main aims: (1) to gain physician’s first-hand accounts of their current needs and barriers in providing chronic pain care to patients in British Columbia (BC), Canada, and (2) to assess their current use, interest, and perceived barriers to employing eHealth technology. Recent investigations into healthcare provider perspectives on chronic pain management and eHealth use in Canada have tended to focus on a specific pain condition (e.g., cancer [59], fibromyalgia [60], knee osteoarthritis [61]), domain of pain care (e.g., training [62], therapeutic cannabis [63], opioid prescribing [32, 64, 65], tele-mentoring [66]), or health profession (e.g., rheumatologists [67], pharmacists [68], and physician assistants [55]), with data collection occurring prior to the COVID-19 pandemic.

The current research aimed to take a wider lens by employing a cross-sectional survey design to assess physicians’ knowledge and barriers in the delivery of pain care, as well as practices and attitudes towards eHealth technologies. We aimed to capture diverse perspectives by recruiting physicians practicing across a broad spectrum of healthcare settings (i.e., primary care, pain specialty clinics, hospitals), in both rural and urban areas. Further, by collecting data approximately one year into the COVID-19 pandemic, we sought to capture views at a peak time for both changes in the delivery of pain care and the need for eHealth. The overarching goal was to contribute to the ongoing efforts to offer more effective pain care in Canada by elucidating current practices, needs, and barriers in the delivery of pain management and implementation of eHealth technology.

## Method

### Recruitment

A convenience sample of 100 physicians licensed to practice in BC were recruited to participate in an online survey about barriers in the management of chronic pain, as well as their current usage of, and attitudes towards eHealth technology. As done in similar research, a sample size of 100 was chosen to gather a preliminary overview of physicians’ perspectives given funding limitations [69, 70]. We attempted to obtain a broad range of perspectives by emailing a brief study description to pain- and medical-related organizations (distributed across health authorities and geographical regions in BC), along with an email invitation and social media advertisement. Interested organizations could forward the email invitations to eligible individuals within their network and/or share the advertisement on their social media pages. A detailed description of the study was housed on the Thrive Health website and this description contained a link to the online consent form and survey questions for interested participants. To be eligible, participants were required to be: (a) a physician, (b) licensed and practicing in BC, Canada, and (c) treating chronic pain as part of their practice, to at least some degree. There were no exclusion criteria. Only participants meeting the eligibility criteria were directed to the survey. Due to funding constraints for participant honoraria, data collection was halted once a sample of 100 was achieved.

### Procedure

Qualtrics was used to obtain informed consent and to administer the survey questions. Participants could opt to receive a $40.00 honorarium or to have it donated on their behalf to a pain- or mental health-related non-profit agency. Ethics approval for this study was obtained by the University of British Columbia Okanagan Behavioural Research Ethics Board (H20-03701) and informed consent was obtained from all participants prior to taking part. Further, all methods were carried out in accordance with relevant guidelines and regulations. Study recruitment and data collection took place from April 01, 2021 to May 31, 2021.

### Survey

The online survey was organized into four main sections: (1) demographic and practice characteristics (e.g., years of practice, specialty), (2) chronic pain practice and educational support needs, (3) perceived barriers to providing chronic pain management in their practice (e.g., lack of time, fear of prescribing opioids), and (4) use of, and attitudes towards, eHealth technology. Regarding eHealth technology, we were specifically interested in physicians’ use of electronic platforms for collecting patient-reported intake and outcome data, barriers to uptake, and interest in specific eHealth platform features. The survey questions were developed by the study authors (psychologists, pain providers, and e-health specialists) based on their clinical experience and a review of existing literature on chronic pain care and eHealth technology. Questions were tailored to address prominent issues facing physicians, including physician workload and burnout [35], an increased prevalence of chronic pain and mental health comorbidities amidst the pandemic [38], the Canadian opioid crisis [71], and an ongoing demand for greater patient-centred pain care [3]. The majority of questions were quantitative with multiple-choice or Likert-scale response formats. Participants were also given the opportunity to elaborate on their perceived barriers or needs via an open-ended question (i.e., “If there is anything else you would like us to know, please share your comments below [e.g., other barriers you may experience with pain management and technology]”). This question was designed to give participants an opportunity to elaborate on their answers, as well as provide comments on barriers that may not have been captured in the structured response sections. The survey took approximately 10–15 min to complete.

### Analysis

Survey responses were summarized using descriptive statistics (i.e., frequencies and percentages for categorical variables and means with standard deviation for continuous variables). Preliminary descriptive analyses revealed no notable differences in the pattern of responses based on key demographic (gender, age) or practice (rural/urban, specialty) factors. Thus, results were aggregated across the entire sample. All analyses were performed in SPSS v27. Responses to the final open-ended question were analyzed using conceptual content analysis to identify prominent barriers in pain care and eHealth technology that may not have been captured in the closed-ended questions [72, 73]. Preliminary codes were derived inductively by the first and third authors and then expanded and contracted to best fit the data via an iterative process with the last author. This resulted in eight main content categories.

## Results

### Demographic and practice characteristics

A total of 100 physicians in BC participated in this study. Demographic and practice characteristics for the sample are presented in Table 1. The majority of participants were female (61%) and the largest age group was 30–39 years (35%) - representing a slightly younger and more female sample than licensed physicians in BC [74]. The sample included physicians practicing in rural and urban areas (rural = 48%, urban = 42%; both = 10%) across the five BC health authorities. When asked what area best describes their practice, most physicians selected primary and urgent care (82%), followed by medical specialty (9%; e.g., internal, neurology, oncology, anaesthesia), surgical specialty (5%), and mental health specialty (4%; e.g., psychiatry, addictions). Just over half of the sample (52%) reported working in more than one location, with family practice (72%) and hospital (49%) being the most common. Participants ranged in years of experience as a physician from 1 year to 50 years (M = 16.47 years, SD = 12.81).

**Table 1:** Demographic and Practice Characteristics of Physicians

Characteristic	Respondents, %
**Gender**	
Male	39
Female	61
**Age**	
20–29	3
30–39	35
40–49	26
50–59	18
60–69	15
70+	3
**Practice Setting**	
Rural	48
Urban	42
Both	10
**Specialty**	
Primary and Urgent Care	82
Medical Specialty	9
Surgical Specialty	5
Mental Health Specialty	4
**Practice Location** ^**a**^	
Family Practice	72
Interdisciplinary Pain Clinic (Private)	5
Interdisciplinary Pain Clinic (Public)	6
Walk-in Medical Clinic	14
Urgent Primary Care Center	7
Hospital	49
Other	19

Note. Percent reported without frequency as N = 100

^a^ More than one location could be selected for this question

### Chronic pain practice, knowledge, and educational support needs

Most of the sample reported that their practice involved either “some” (72%) or “a large portion” (20%) of chronic pain management. Physicians felt that treating chronic pain completely (26%), mostly (35%), somewhat (36%) or slightly (3%) falls under their scope of practice or expected role. Additionally, they rated their knowledge of assessing and treating chronic pain as expert (8%), very good (23%), average (51%), fair (12%), or limited (6%).

From a list of options, physicians were asked to identify which areas they could benefit from additional information or education related to pain management. The vast majority (88%) indicated they would benefit from more information surrounding community-based resources for people living with chronic pain. Participants also indicated a need for additional education in the following areas: non-pharmacological treatment options for pain (e.g., psychotherapy, physiotherapy; 59%), alternative pharmacological treatment options for pain (e.g., cannabis; 56%), safe prescribing practices/guidelines for opioids in pain management (34%), and “other” (15%). Only 3% of participants indicated that they would not benefit from any additional information or education.

### Perceived barriers and needs for pain management

Next, physicians were asked to indicate the frequency with which they experienced a variety of barriers in delivering pain care (see Table 2). Within the category of infrastructure, the most common barrier was a lack of interdisciplinary team support, with 56.3% of physicians indicating this was “always” experienced. Regarding clinical assessment, the most frequently experienced barriers pertained to a lack of time. Specifically, about one-third of physicians reported that they “always” lack time to review and discuss patients’ intake responses (32.9%) and engage in shared decision-making (30.3%). When asked about clinical treatment barriers, half the sample (50.5%) reported “always” experiencing difficulty identifying community pain resources for referral (e.g., education, support groups, therapy).

**Table 2:** Perceived Barriers to Chronic Pain Management

Barrier	Always n (%)	Sometimes n (%)	Never n (%)
**Infrastructure**			
Lack of interdisciplinary team support	54 (56.3%)	36 (37.5%)	6 (6.3%)
Difficulty providing care to rural patients due to distance	21 (28.0%)	44 (58.7%)	10 (13.3%)
Lack of receiving patient data from other healthcare providers	17 (19.8%)	58 (67.4%)	11 (12.8%)
Difficulty prioritizing patients from waitlist	15 (21.4%)	28 (40.0%)	27 (38.6%)
**Clinical Assessment**			
Lack of time for shared decision making with patient	30 (30.3%)	51 (51.5%)	18 (18.2%)
Lack of time to review and discussing patients’ responses on intake form during visit	26 (32.9%)	39 (49.4%)	14 (17.7%)
Lack of time to assess patient reported outcomes	25 (27.8%)	51 (56.7%)	14 (15.6%)
Difficulty manually scoring questionnaire results	9 (14.5%)	29 (46.8%)	24 (38.7%)
Difficulty assessing patient risk for opioid abuse	7 (7.4%)	73 (76.8%)	15 (15.8%)
Difficulty building trust with patients	4 (4.0%)	77 (77.8%)	18 (18.2%)
**Clinical Treatment**			
Difficulty identifying community pain resources for referral	50 (50.5%)	44 (44.4%)	5 (5.1%)
Difficulty managing co-occurring mental health conditions	32 (32.0%)	61 (61.0%)	7 (7.0%)
Difficulty having conversations regarding medication abuse	27 (29.0%)	38 (40.9%)	28 (30.1%)
Fear of prescribing opioids	12 (12.0%)	79 (79.0%)	9 (9.0%)
Difficulty making decisions about whether patient would or would not benefit from a specific treatment plan	10 (10.0%)	69 (69.0%)	21 (21.0%)

Note. Physicians could select “not applicable” for any barrier that did not apply to their practice (not shown here for simplicity). The percent displayed is based only on the number of respondents for whom the barrier was applicable; consequently, raw scores in each row may not sum to 100

A subset of participants (43%) responded to an open-ended probe for additional comments related to perceived barriers in pain care and e-Health technology. Table 3 presents eight categories of barriers and needs that were identified based on the data, along with representative quotes. While the themes derived from the open-ended responses were generally consistent with the most significant barriers identified in the closed-ended questions, such as the need for enhanced patient access to allied health professionals and non-pharmacological treatments, some additional barriers and preferences for eHealth technology were also highlighted. Specifically, the closed-ended questions did not fully capture the demand for eHealth technology with integrative features, as well as the concerns expressed by some physicians regarding access to technology and the internet.

**Table 3:** Physician-reported barriers and needs to improve pain management

Categories	N	Representative Quotes
**Access to Allied Health Professionals and Non-Pharmacological Treatment**	15	“The lack of coverage for allied health (e.g., psychotherapy, physiotherapy, massage therapy) creates significant barriers to other non-medication modalities.” “[The] biggest barrier is lack of access/financial support for patients requiring physical and psychological treatments”
**eHealth Platform with Integrative Features**	10	“We don’t have capability for patient questionnaires/forms to be electronically entered into our EMR. Currently, they [patients] have to fill the form out on paper and then the form has to be scanned into our EMR and the data has to be manually entered in order for it to be tracked. Very time consuming and thus we tend not to use the forms very much as not easy to track data generated from the forms.” “I’d like app integration that asks for pain scores and complications post procedure rather than waiting 2 weeks to find out their pain was worse for 72 h after procedure or they went to the ED [Emergency Department] instead of calling me.”
**Access to Pain Specialists and Multidisciplinary Pain Clinics**	9	“The main barrier in BC is the complete lack of multidisciplinary chronic pain programs. There is a hodgepodge of programs that offer limited options (and often just short-term) for patients and their primary care providers.” “Lack of inter-professional (ie Team) supports in rural areas is a real challenge.”
**Improved Support for Opioid Prescribing and Management**	7	“I regard the restrictions placed by CPSBC [College of physicians and Surgeons of BC] – with inevitable audits for prescribing opioids – as significant deterrents to assuming care of patients with chronic pain on opioids.” “Over the past 5 years, the prescribing of opiates has been questioned/advised against to the extent that I feel that I am a bad doctor to prescribe them for patients whose pain is not controlled with prescription nsaids (if they can take them) and acetaminophen. The culture of the [CPSBC] and in the medical community is now that one is an “outlier” if one prescribes them for patients. I struggle with this, as I know that there are some situations that patients need narcotics, and untreated chronic pain has mental health consequences.”
**Improved Links to Community Resources to Community Resources**	4	“What is lacking for me is access to community resources to dovetail the patient to when they’re discharged from hospital.” “A high-quality list of community resources for different types of pain would be very useful as part of a technological option. (e.g. could look with a patient on a map and filter types of supports).”
**Improved Remuneration for Physician Time**	4	“In primary care, the fee for service model runs on a 7–10 min appointment expectation, which does not allow for good chronic pain care beyond basic interventions.” “Physicians need to be taught how to manage chronic pain - and remunerated adequately for it, as it is very time consuming and often involves challenging conversations and patients who have suffered and do not trust the system, making it more challenging to connect with them.”
**Improved Pain Education for Physicians and General Public**	4	“Management of NCCP [Noncancerous chronic pain] is not taught in med school.” “I believe more public education is needed to change the broader societal understanding of pain and expectation of the process and management.”
**Patient Access to Internet/Technology**	4	“My main barriers: low-income patients who have limited/no access to technology…” “Some patients are not comfortable or equipped to work online or may have poor internet connections”

Note. Themes were extracted from responses to an open-ended question: “If there is anything else you would like us to know, please share your comments below (e.g., other barriers you may experience with pain management and technology).” N = 43

### eHealth Technology

Physicians were asked how often per week they use eHealth technology for pain management in their practice. As shown in Table 4, the most frequently used technology was Electronic Medical Records (EMR; Several times a day = 90%), followed by virtual patient visits (Several times a day = 74%). In contrast, the majority of the sample reported never using eHealth technology for remote patient monitoring (74%) or mobile patient apps (53%) in their practice.

**Table 4:** Frequency of eHealth technology use among physicians (N = 100)

eHealth Technology	Several Times a Day	Once a Day	Several Times a Week	Once or Twice a Week	Never
**Electronic Medical Record (EMR)**	90	0	5	1	4
**Virtual Patient Visits**	74	2	10	8	6
**Electronic Health Record (EHR)**	53	4	13	8	22
**Physician-Physician Consultations**	16	10	31	40	3
**Remote patient monitoring**	4	1	6	15	74
**Mobile health apps (for patient use)**	3	3	15	26	53

Table 5 presents physicians’ current use of eHealth technology for the collection of patient data, as well as barriers to the uptake of this technology. Only a small proportion of physicians reported currently using electronic methods to collect patient intake information (21%) and patient-reported outcomes (PROs; 14%). Over half of physicians indicated that they were either undecided or uninterested in this type of technology, and this was most commonly because “the implementation was too complicated.” The most cited “other reason” for being undecided/uninterested was being unaware of, or having limited knowledge of, the available options (Intake = 7; PROs = 16). Other commonly reported reasons included: preference for in-person assessment, technology not offered at their clinic, technology or internet concerns for patients, and concern around data quality or usefulness, respectively.

**Table 5:** Use of Technology for Chronic Pain Management

Question and Answer	N	%
**Do you use electronic methods to collect intake information for new patients? (N = 100)**		
Yes, I am currently using it	21	21.0%
No, but I plan to in the future	28	28.0%
No, not currently or in the future	35	35.0%
Undecided	16	16.0%
**Why not/undecided? (N = 51)** ^a,b^		
Software too expensive	9	17.6%
Implementation too complicated	19	37.3%
Patients prefer paper	4	7.8%
No proven benefit	3	5.9%
No need	7	13.7%
Other reason	27	52.9%
**Do you use electronic methods to collect patient-reported outcomes (PROs)? (N = 100)**		
Yes, I am currently using it	14	14.0%
No, but I plan to in the future	29	29.0%
No, not currently or in the future	36	36.0%
Undecided	21	21.0%
**Why not/undecided? (N = 57)** ^a,b^		
Software too expensive	9	15.8%
Implementation too complicated	26	45.6%
Patients prefer paper	4	7.0%
No proven benefit	2	3.5%
No need	6	10.5%
Other reason^c^	30	52.6%

Note. Percent calculated based on the number of participants who responded to that question, as indicated by N

^a^ Question only shown to the subset of participants who responded “No, not currently or in the future” or “Undecided” to the previous question

^b^ More than one reason could be selected

^c^ Three of these respondents did not provide a specified other reason

**Fig. 1:**
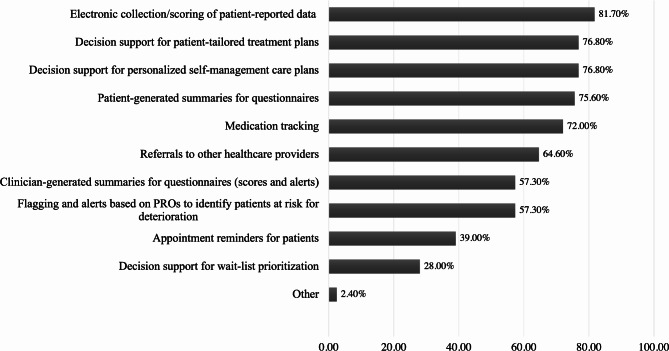
Preference for eHealth Feature. This question was only shown to participants who indicated they would be interested in an eHealth platform, based on their response to a previous question. Percent based on the number of participants who responded. More than one feature could be selected. N = 82

Next, physicians were asked if they were interested in an electronic platform that could offer such capabilities as automated collection of patient data, smart triage and decision support, and personalized care plans. Participants who responded yes (82%) were then asked which specific features they would prefer from a selection of choices. As shown in Fig. 1, many of the features were of interest to physicians. The most desired feature was the electronic collection and scoring of patient-reported data (81.7%), followed closely by decision support for patient-tailored treatment plans (76.8%) and self-management care plans (76.8%), as well as patient-generated summaries for questionnaires (75.6%).

## Discussion

Chronic pain is a highly prevalent and disabling chronic condition, which requires an interdisciplinary approach to optimize patient functioning and wellbeing [15, 42, 75]. With the onset of COVID-19, there have been substantial challenges to the delivery of chronic pain care and an increased need for eHealth. Given this considerable demand and shift in practice, we surveyed 100 BC physicians to identify current barriers and needs to support the provision of chronic pain care, and to assess the use and interest in eHealth technologies within the context of the COVID-19 pandemic. The most prominent barriers that emerged were a lack of access to interdisciplinary treatment and allied health support, challenges related to opioid prescribing and management, and a lack of time to manage the complexities of chronic pain. Although the vast majority of physicians expressed interest in employing a diverse array of eHealth technologies, the present findings suggest that several barriers to implementation remain, with very few providers expanding beyond virtual visits and electronic medical records in their daily practice.

### Interdisciplinary pain support and pain education

Over 90% of surveyed physicians reported a lack of interdisciplinary team support, difficulty identifying community pain resources, and challenges managing patients with co-occurring mental health conditions. Additionally, nearly all (88%) physicians indicated a need for more information on community-based pain resources for their patients. These concerns were echoed and elaborated upon in the free-response portion, with more than half of physicians expressing a lack of patient access to pain specialists, allied health professionals, and non-pharmacological treatment options. Physicians largely attributed these problems to a shortage of service providers, long waitlists, and a lack of public funding for allied health services.

It should be noted that while these barriers are not new, the consequences of limited access to, and knowledge of, community resources and interdisciplinary services may be particularly detrimental in the context of the COVID-19 pandemic. During the pandemic, individuals are experiencing numerous psychosocial stressors including increased social isolation, restricted physical activity, and financial stress, among other challenges [39, 42, 76]. These stressors are likely to exacerbate the psychosocial comorbidities associated with chronic pain (e.g., anxiety, depression), making access to interdisciplinary pain care and a biopsychological approach even more crucial [39, 62].

### Opioid prescribing and management

Concerns with prescribing opioids (sometimes or always = 91%) and difficulty assessing risk for opioid abuse (sometimes or always = 80%) were also endorsed by most of the sample. These concerns were elaborated on in the open-ended responses, with several physicians expressing a need for more comprehensive guidelines that allow for clinical judgement and flexibility. Due to the perceived restrictiveness and ambiguity of the guidelines, physicians expressed fear of being audited, hesitancy to take on patients with opioid use, and challenges tapering doses. Barriers pertaining to vague guidelines and fear of sanctions for prescribing opioids are consistent with the concerns that emerged in a recent qualitative study of primary care providers in Ontario, Canada [33]. Current guidelines suggest that interdisciplinary support should be offered for patients who experience difficulty with tapering/cessation [77, 78]. Yet, as conveyed in the present research, there is a scarcity of interdisciplinary pain clinics and affordable allied health options to support opioid tapering/cessation. To achieve equitable delivery of pain care in the midst of the opioid crisis, physicians require clear and supportive protocols, along with improved knowledge for non-pharmacological alternatives [33]. The high prevalence of these concerns is not surprising, in light of not only an opioid epidemic but also the COVID-19 pandemic. Indeed, opioid use has been linked to worsened outcomes for COVID-19 patients [79] and there has been a surge in opioid-related abuse and overdose deaths, further complicating prescribing decisions [80, 81].

### Lack of time

Regarding clinical assessment of chronic pain, the most frequently endorsed barriers pertained to lack of time. Over 80% of physicians reported sometimes or always experiencing a lack of time for shared decision-making with patients, as well as a lack of time to review and discuss patient-reported data (i.e., intake measures and PROs). This barrier was also reflected in the open-ended responses, with physicians expressing that lack of time and remuneration for appointments reduced their ability to assess and manage the complexities of chronic pain.

Although this barrier of time is not unique to chronic pain, when combined with the high demand (e.g., frequent and complex visits) and perceived lack of support/knowledge in pain management, insufficient time likely places a considerable strain on primary care providers and their ability to effectively deliver pain care. Further, as previously noted, with the pandemic we have seen a restriction of healthcare services, increased clinic volumes, and higher rates of chronic pain placing further demand on providers’ already limited time [41, 42]. In addition to adding more primary care providers to the system, another possible way to help alleviate this time pressure is to adjust the funding model. For example, BC has already introduced financial incentives for primary care providers who treat patients with chronic illnesses such as diabetes and hypertension [82–84] and this could be expanded to include chronic pain.

### eHealth

A total of 82% of participants expressed interest in adopting an eHealth platform to assist with pain care, with the greatest interest in technology for automated collection and scoring of patient-reported measures, decision support, patient-generated summaries, medication tracking, and referrals to community-based providers. These features could help alleviate several of the barriers to pain care raised in the present study. For example, by automating certain clinical tasks, physicians can redirect their limited time to other aspects of patient care [85–87]. One example of the successful automation of clinical tasks is the Collaborative Health Outcomes Information Registry (CHOIR; https://choir.stanford.edu) system - a web-based application used actively and widely in the United States to track, monitor, and visualize health outcomes for patients with chronic pain.

Similarly, for providers practicing in low-resource, rural and remote areas, eHealth technology can offer unique benefits that may address several other barriers raised in the present study (e.g., lack of interdisciplinary support, limited pain knowledge) [88]. For instance, Project ECHO™ is a knowledge-sharing network for pain providers initiated in Ontario, Canada and has recently expanded to other provinces. Specifically, clinical experts are connected with primary care providers through telehealth technology to share best practices in pain management, overcome geographical barriers to education, and increase providers’ competency and confidence in treating chronic pain [88–90]. Participation by healthcare providers in Project ECHO™ is associated with improvements in knowledge regarding chronic pain assessment, treatment practices, and opioid prescribing [66, 90].

The COVID-19 pandemic has rapidly expanded interest in, and utilization of, virtual care [91–93]. Yet low adoption of a range of eHealth technologies was found in the present study. For example, despite great interest in remote collection of patient-reported intake and outcome data, only 21% and 14% of physicians endorsed current use of these technologies, respectively. This low adoption rate is in stark contrast to the rapid uptake of virtual patient-visits [58]. In the present study, physicians frequently indicated that the software was too complicated, too expensive, and/or they were unaware of the options available to them. These concerns may be partially attributable to physicians receiving little to no formal education on eHealth technology during medical school [94, 95]. Moreover, several physicians stressed that any eHealth platform that collects patient intake and outcome data would need to integrate with EMR for successful implementation into their practice. As such, improved design, awareness, funding, and training are required to achieve successful implementation of eHealth technology in routine practice [96]. Indeed, as advocated by Houwink et al. [97], primary care providers need to be “supported, educated, and involved in all processes, from the development of effective eHealth solutions to their implementation in regular care” (pp.109).

Lastly, the findings offer a reminder that modified or non-technology options are still required for certain patient populations, such as those with cognitive limitations or without internet access. For example, in Canada, there are large variations in who has internet access [98]. Specifically, the most recent Canadian data indicate that 98.6% of households in urban areas are able to access broadband internet services, compared to just 45.6% in rural households and 34.8% in First Nation reserves [99]. Despite these realities, eHealth continues to show promise in closing the gaps in access to health care and improving physician throughput.

### Strengths, limitations and future directions

Although participants in the current study were mostly primary and urgent care practitioners, this reflects the physician population most often responsible for chronic pain management in Canada. Moreover, we captured diverse perspectives by recruiting rural and urban physicians practicing in a range of healthcare settings across the province, with varying degrees of chronic pain experience and knowledge. Nonetheless, the nature of our sample precluded any formal statistical comparisons of perceived barriers between practice settings, health authorities, and areas of specialty. This requires attention in future research. Additionally, although a strength of this research, data collection occurred approximately one year into the COVID-19 pandemic and, as such, perspectives and eHealth adoption rates may change as the threat and impact of COVID-19 shifts over time. Last, a limitation to consider is that we did not provide a specific definition of interdisciplinary care to survey participants. As a result, we cannot be certain about how participants interpreted the term when expressing a need for more interdisciplinary support. It is possible that some participants used the term interchangeably with multidisciplinary care, which involves healthcare professionals working independently within their respective specialties. In contrast, interdisciplinary care involves healthcare professionals working collaboratively as a team to provide comprehensive care. Despite this limitation, the open-ended responses from physicians helped us gain a better understanding of the issue, and we found that many participants expressed the need for additional community-based resources and mental health support. Future research should continue to explore physician experiences within and outside of Canada, and include the perspective of allied health practitioners who also see a large proportion of people with chronic pain in their practice.

## Conclusions

This study examined the current practices, knowledge, barriers, and preferences for chronic pain management and eHealth technology during the COVID-19 pandemic in a sample of physicians in BC, Canada. The most consistent and compelling message that emerged from participants was that physicians are overwhelmingly challenged by a lack of referral options for their chronic pain patients, including interdisciplinary programs, pain specialists, and allied health support. These findings consolidate calls for the urgent need of a multi-pronged strategy that links patients with accessible, affordable, and empirically-supported treatments that address pain from a biopsychosocial approach [75]. Importantly, improved access and coverage to non-pharmacological and non-physician pain treatment options would require changes to insurance plans and government healthcare policies [22, 100].

Additionally, despite the promise that eHealth technology holds for addressing several of the current barriers identified, particularly amidst the COVID-19 pandemic, adoption rates remain low. Improved design, awareness, funding, and training are required to achieve successful implementation of eHealth technology [98] . As we move through the fourth year of the COVID-19 pandemic, there exists no better time for governments, front-line workers and software developers to work collaboratively to determine how to best integrate eHealth tools into standard practice.

## Data Availability

The survey and dataset(s) supporting the conclusions of this article are available at https://osf.io/cqd7j.

## Abbreviations

BC: British Columbia
CI: Confidence Interval
EMR: Electronic Medical Record
PROs: Patient Reported Outcomes
SMA: Shared Medical Appointments
